# Recurrent massive hemoptysis in a patient with cystic fibrosis: balloon assisted Onyx embolization after bronchial artery coil recanalization

**DOI:** 10.1186/s42155-020-00200-8

**Published:** 2021-01-05

**Authors:** Raghav R. Mattay, Richard Shlansky-Goldberg, Bryan A. Pukenas

**Affiliations:** Department of Radiology, Hospital of the University of Pennsylvania, 3400 Spruce Street, Philadelphia, PA 19104 USA

**Keywords:** Bronchial artery embolization, Balloon catheter, Onyx, Cystic fibrosis

## Abstract

**Background:**

Although not standard of care, Cystic Fibrosis patients with recurrent hemoptysis occasionally have coil embolization of bronchial arteries. In the event of recanalization of these arteries in this specific subset of patients, the presence of indwelling coils makes the prospect of conventional particle embolization more difficult, preventing both adequate catheterization of the coiled segment and reflux of the particles.

**Case presentation:**

In this report, we describe a case of bronchial artery embolization of a complex Cystic Fibrosis patient with massive hemoptysis from recanalized coiled bronchial arteries utilizing a Scepter Balloon Catheter® (Microvention Terumo, USA) in administration of the liquid embolic agent Onyx® (Medtronic, USA).

**Conclusions:**

The Scepter occlusion balloon catheter allowed for careful placement of the tip within the interstices of the pre-existing coils, allowing for Onyx injection directly into the coil mass without reflux, reconfirming the benefits of Onyx embolization in bronchial artery embolization and providing evidence that the Scepter occlusion balloon catheter should be added to the armamentarium of devices used in complex bronchial artery embolization for Cystic Fibrosis patients with massive hemoptysis.

## Background

Intercostal and bronchial artery embolization with particles is a commonly used procedure to treat massive hemoptysis in patients with Cystic Fibrosis (CF). Occasionally, while not standard of care, coils may be used but prevent repeat embolization when needed. Patients with coils and recurrent hemoptysis may have recanalized arteries making the prospect of conventional particle embolization more difficult due to inability to safely catheterize the coiled segment and retreatment. Liquid embolic agents such as Onyx® (Medtronic, USA), often used in neuro-interventional procedures (Brassel and Meila 2015), have been shown to be effective in some cases of refractory massive hemoptysis in patients with CF where conventional particle embolization has failed (Ao et al. 2013). In certain situations, the injection of Onyx through a microcatheter can be technically challenging, with resultant complications including Onyx reflux and non-target embolization. In order to mitigate complications, a balloon catheter can be used to augment flow of Onyx (Paramasivam et al. 2013). We describe our experience of administration of liquid Onyx through a Scepter® XC balloon catheter (Microvention Terumo, USA) into a recanalized intercostal artery with indwelling coils to successfully treat a patient with refractory massive hemoptysis.

## Case presentation

The patient was a 23 year-old Caucasian female with a history of CF and persistent hemoptysis, preventing her from being able to lie supine. She presented for semi-emergent bronchial artery embolization treatment for her hemoptysis. The patient had undergone coil embolization previously for the same condition at an outside hospital.

Preoperative examination revealed no contraindications to bronchial artery embolization and patient was placed under conscious sedation throughout the procedure. A 5-French Cobra catheter was placed in the descending aorta through the femoral artery using standard Seldinger technique.

**Fig. 1:**
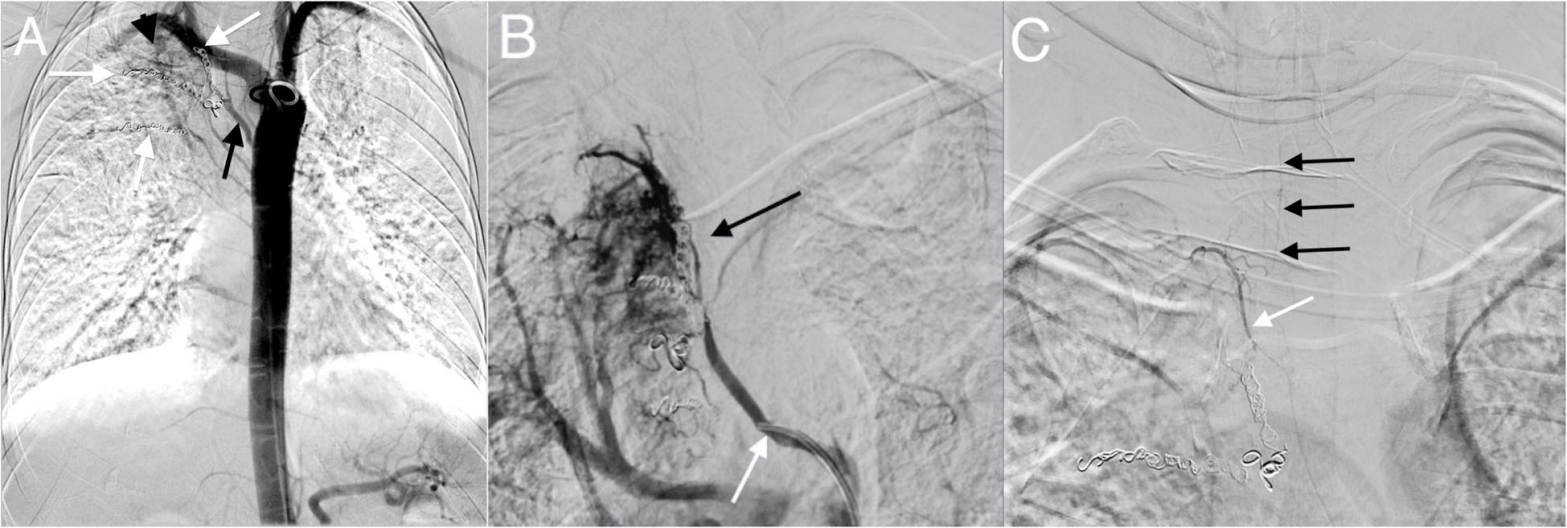
**a** Thoracic AP digital subtraction aortogram shows a dominant right intercostal bronchial trunk (black arrow) and additional hypertrophied right intercostal arteries, all of which had been previously embolized with coils (white arrows). Tortuous ectatic vessels are also present arising from the upper thoracic segmental arteries (black arrowhead). **b** Magnified right anterior oblique (RAO) superselective angiogram with microcatheter in the right fourth intercostal artery (white arrow) demonstrates multiple recanalized coils (black arrow). **c** Slight RAO superselective angiogram of a branch of the prominent intercostal bronchial trunk (white arrow) showed a spinal artery branch (black arrows). This bronchial artery was not embolized

The diagnostic portion of the examination via conventional contrast angiography demonstrated tortuous and ectatic vessels from several bronchial arteries perfusing the upper lobe of the right lung, and right bronchial to pulmonary arterial shunting (Fig. 1a). The fourth intercostal artery was selectively catheterized with a diagnostic catheter and a superselective angiogram demonstrated multiple recanalized coils in the feeding artery (Fig. 1b). Additionally a prominent spinal artery originating from the bronchial artery was detected (Fig. 1c).

**Fig. 2:**
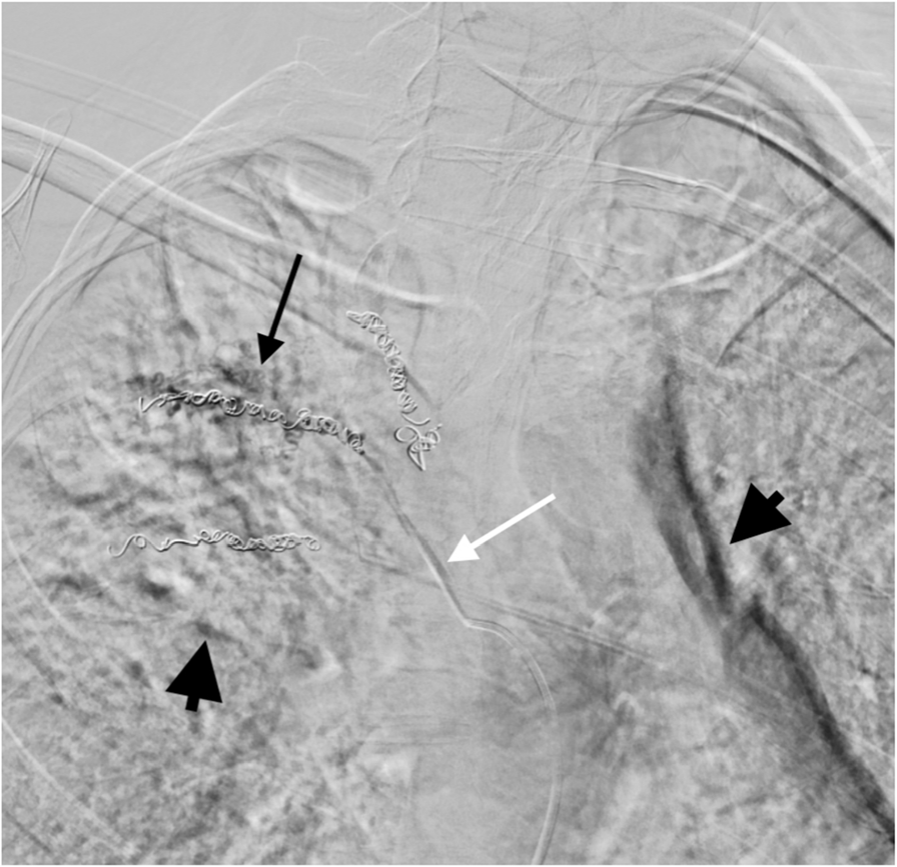
Slight RAO projection of digital subtraction arteriogram during attempted microparticle infusion with evidence of reflux along the microcatheter located in the fourth intercostal artery (white arrow). There is some minor, incomplete filling of the tortuous, ectatic vessels distal to the coils (black arrow). Large black arrowheads demonstrate breathing artifact from digital subtraction angiography

**Fig. 3:**
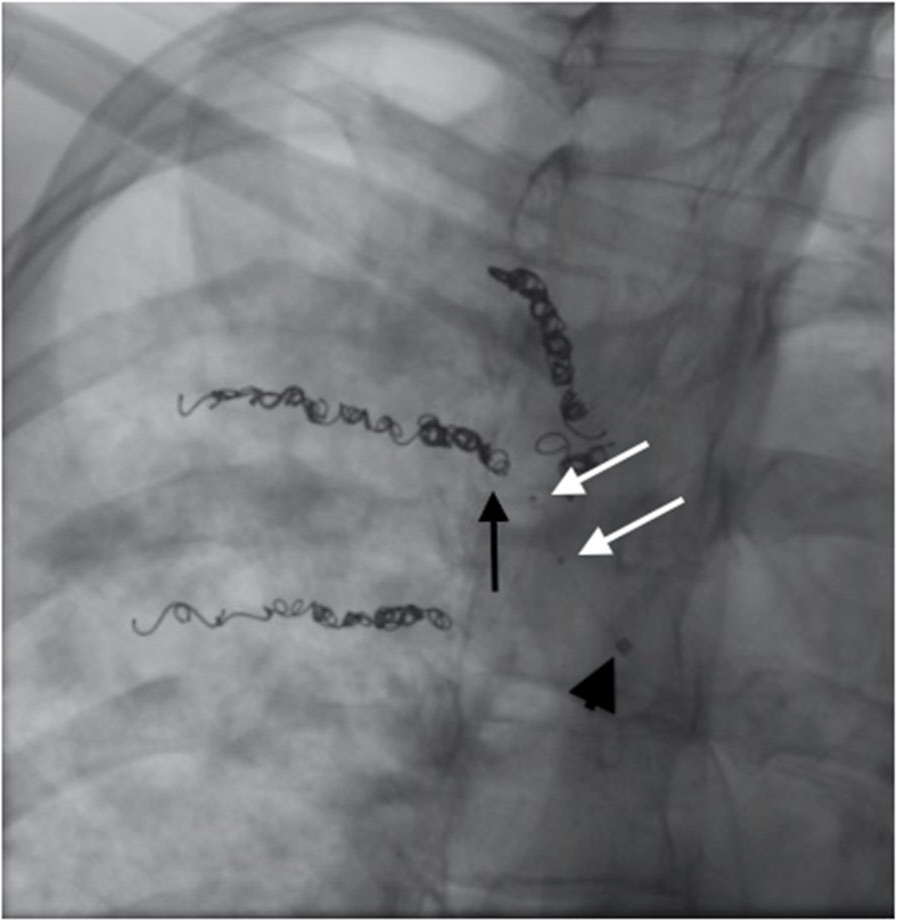
Magnified slight RAO fluoroscopic image demonstrating an uninflated 4 × 11 mm Scepter XC balloon catheter with its tip (black arrow) within the interstices of the coil mass. White arrows point to the proximal and distal radio-opaque markers showing the location of the uninflated balloon of the Scepter XC balloon catheter. The black arrowhead shows the radio-opaque distal tip of the Neuron 6F 053 Delivery Catheter® (Penumbra, USA)

Conventional particle embolization was attempted, but the indwelling coils prevented complete filling of the tortuous, ectatic vessels distal to the coils and caused reflux of the particles (Fig. 2). A decision was then made to embolize this branch with Onyx 18. To facilitate this, the 5-French Cobra catheter was replaced with a Neuron 053® guide catheter (Penumbra, USA), which was positioned into the intercostal artery. A 4 × 11 mm Scepter occlusion balloon microcatheter was then prepared using standard technique and positioned with its tip within the interstices of the coil mass (Fig. 3).

**Fig. 4:**
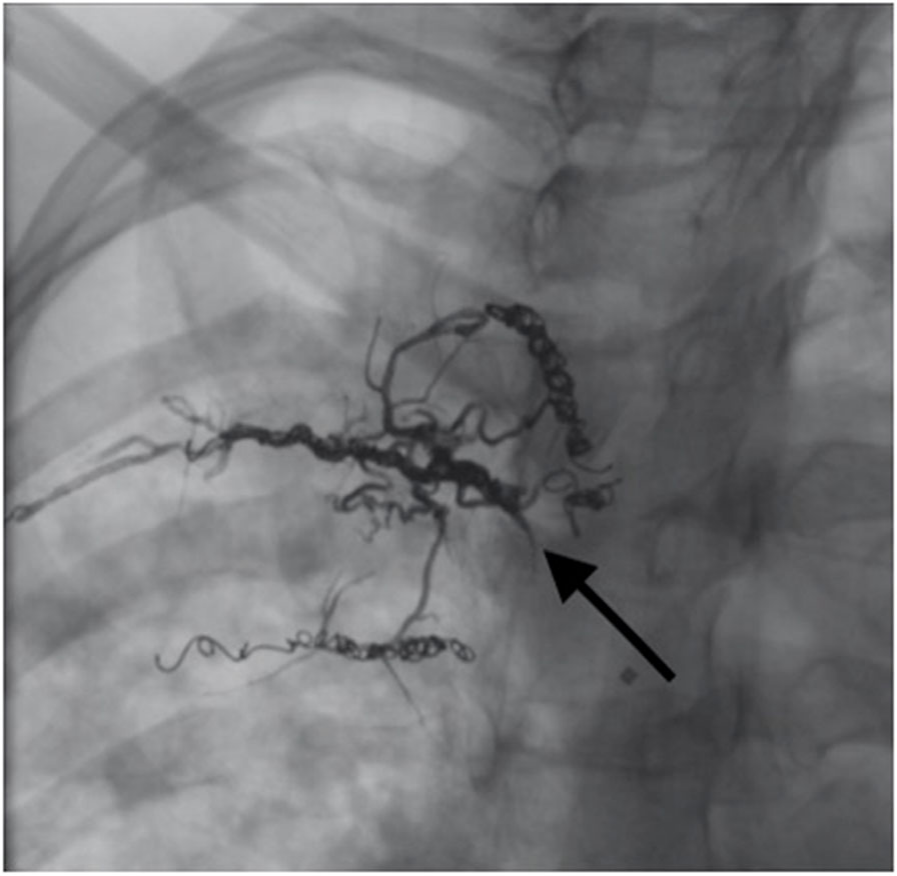
Magnified slight RAO fluoroscopic image demonstrating adequate filling of the feeding pedicle and multiple collateral vessels with radio-opaque Onyx

The catheter was then purged with Dimethyl sulfoxide (DMSO). Following DMSO injection, the balloon was inflated and Onyx 18, using common preparation technique (Guimaraes and Wooster 2011), was injected under continuous fluoroscopic guidance. Onyx was injected through the existing coil mass until there was filling adequate filling of the feeding pedicle and multiple collateral vessels within the lung (Fig. 4). Careful inspection of both roadmap and native (non-subtracted) imaging was performed during slow Onyx injection to ensure adequate penetration into the ectatic vessels without proximal reflux into the parent intercostal artery. The balloon was inflated and Onyx was injected for approximately 14 min. During Onyx injection, the patient reported mild pain that quickly resolved, without further intervention.

**Fig. 5:**
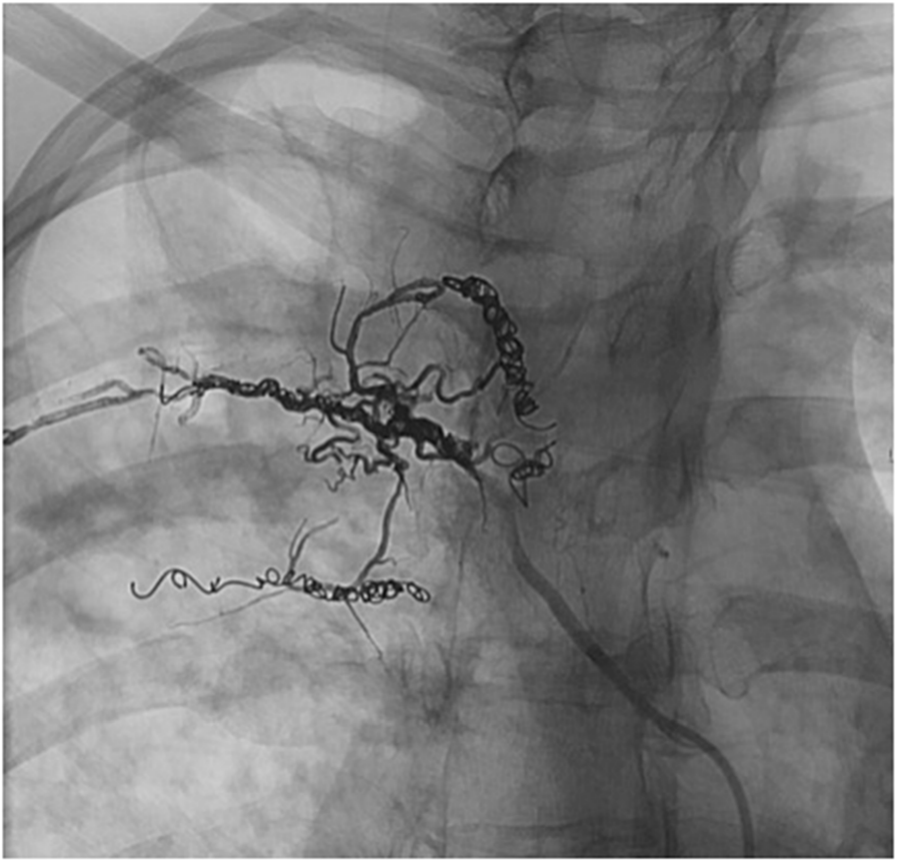
Superselective slight RAO postembolization arteriography demonstrated no residual flow to the diseased portion of the lung from the targeted feeding pedicle

At this point, the balloon catheter was deflated by aspirating the balloon port and removed uneventfully. Postembolization arteriography demonstrated no residual flow to the diseased portion of the lung from the targeted feeding pedicle (Fig. 5) and an Onyx cast filling of numerous branches originating from the fourth intercostal bronchial trunk without casting of the pulmonary arteries.

Next, the right fifth intercostal artery was selected and further diagnostic arteriography was carried out. Although coils were present, a proximal branch could be embolized in the conventional fashion with 700–900 microspheres. Post embolization selective arteriography demonstrated no residual flow from the fifth intercostal artery and appropriate pruning of embolized vessels. The catheters and wires were then removed from the patient and hemostasis was achieved using manual compression. Follow up at 6 and 12 months revealed no evidence of further hemoptysis or other complications. The patient later underwent successful bilateral lung transplantation.

## Conclusions

Hemoptysis is a common complication of CF, and occurs in approximately 9.1% of patients (Flume et al. 2005). Bronchial artery embolization (BAE) is the recommended procedure for clinically unstable patients with massive hemoptysis according to the Cystic Fibrosis Foundation consensus statement (Schidlow et al. 1993). Despite the effectives of embolization in controlling acute hemoptysis, adults with cystic fibrosis who have undergone BAE for hemoptysis have a much higher risk of respiratory function worsening, death, and need for lung transplantation. (Vidal et al. 2006) Moreover, while a life-saving procedure, recurrence of hemoptysis after BAE is not uncommon and occurs in 18% in those patients with initial life-threatening hemoptysis (Garcia-Olivé et al. 2014) and up to 55% in a cohort of young patients with CF (Barben et al. 2002).

In general, two different types of materials are used in endovascular embolization: solid and liquid agents. Currently, solid agents including Polyvinyl alcohol particles and coils, all lead to mechanical obliteration of the vessel through flow deceleration and subsequent thrombosis (Brassel and Meila 2015; Hurt and Simmonds 2012). The liquid embolic agents include adhesive materials and non-adhesive materials such as alcohol copolymers (Onyx) and n-butyl-2-cyanoacrylate (NBCA) glue. Liquid embolic agents have the advantage of filling a vascular area homogenously and give the operator better control, which decreases the risk and frequency of recanalization when compared to the use of solid embolization materials such as PVA particles. (Brassel and Meila 2015; Pierot et al. 2013). Onyx has been shown to have penetration into capillary vessels as small as 5 μm in diameter (Natarajan et al. 2009), increasing durability and effectiveness for hemoptysis treatment.

Within the realm of BAE, particles or microspheres are the most commonly used embolic agents (Corr 2005; White Jr. 1999). Coil embolization is not recommended and rarely performed for BAE, as presence of coils in the bronchial arteries block future endovascular catheterization and treatment (Monroe et al. 2018), however, coil embolization can be used for short term results. In one study, recurrent hemoptysis from a previously embolized bronchial artery with or without coils occurred a total of 22.2% of the time, noting that approximately a third of these recanalized arteries contained coils (Garcia-Olivé et al. 2014). Similarly, Sagara et al. (Sagara et al. 1998) found an exceedingly high recanalization rate in pulmonary AVMs embolized with coils (57%), furthermore justifying that this embolization agent should be avoided in patients with life-threatening hemoptysis (Sagara et al. 1998), especially in patients with CF who are known to have a higher recurrence rate for hemoptysis.

In this case, the presence of indwelling recanalized coils in the bronchial artery caused reflux of PVA particles during the attempted embolization of the fourth intercostal artery. Since adequate control could not be obtained and superselective catheterization showed multiple tortuous vessels feeding the diseased portion of the right upper lung distal to the coils, a decision was made to embolize with a liquid embolic agent. While other liquid embolic agents such as NBCA are increasingly being used in BAE (Woo et al. 2013; Abdulmalak et al. 2018) with successful results, we chose Onyx for embolization through the Scepter balloon. The inflated Scepter balloon created a “plug” and allowed for more controlled and deeper penetration into distal bronchial artery with indwelling coils without the need of robust forward flow, which is required in the much harder to control NBCA (Urbano et al. 2014). While typically reserved for neurointervention (Linfante and Wakhloo 2007), there have been a few reports of the utility of Onyx in BAE (Ao et al. 2013; Bommart et al. 2012; Adamus Nürnberg et al. 2010; Khalil et al. 2010; Ayx et al. 2017) with low recurrence rates for hemoptysis after embolization. Additionally, BAE with Onyx has few adverse reactions and patients have reported a high tolerance (Adamus Nürnberg et al. 2010; Khalil et al. 2010).

Since Onyx is a liquid agent, a rapid administration may result in reflux and some retrograde embolization, which can compromise normal vasculature and lead to entrapment. Attempting to pull the microcatheter can lead to catheter stretching, rupture, and occasionally rupture of the feeding vessel, causing further hemorrhage (Paramasivam et al. 2013). Development of an Onyx “plug” technique in which a proximal plug is allowed to form at the microcatheter tip before anterograde injection has been shown to allow for prevention of some reflux, however this has shown to lead to increased fluoroscopic times (Weber et al. 2007; Velat et al. 2008).

In this case, in order to minimize reflux of Onyx, a Scepter balloon catheter was used. Since the 1970’s, balloon catheters have been used in neurointerventional procedures allowing for distal fluid delivery while the balloon remains inflated which forms a proximal plug and prevents reflux of the embolization agents (Kerber 1976). More recently Rose et al. showed the utility of balloon occlusion-assisted delivery of Onyx for some peripheral arterial applications including hemorrhage (Rose et al. 2020). The Scepter balloon catheter in particular has a variety of features that makes it very useful in liquid embolization. Firstly, the catheter is DMSO compatible, allowing it to be used with Onyx. Secondly, it has a coaxial double lumen with two corresponding ports, one to inject and the other to inflate the balloon separately. The balloon of the Scepter catheter is also extremely pliable and conforms well to the shape of the vessel and can even herniate into proximal feeders forming a better seal (Paramasivam et al. 2013). The catheter itself is also very flexible and navigable with a 5 mm tip that can be steam shaped to assist in directional control. In this particular example, the Scepter occlusion balloon catheter allowed for careful placement of the tip within the interstices of the pre-existing coils, allowing for Onyx injection directly into the coil mass without reflux.

In this example, we reconfirm the benefits of Onyx embolization in BAE, especially in attempts to embolize recanalized coils. It also provides evidence that the Scepter occlusion balloon catheter should be added to the armamentarium of devices used in complex BAE for CF patients with massive hemoptysis.

## Data Availability

All data generated or analysed during this study are included in this published article.

## Abbreviations

CF: Cystic fibrosis
BAE: Bronchial artery embolization
DMSO: Dimethyl sulfoxide
